# Elevated Peripheral T Helper Cells Are Associated With Atrial Fibrillation in Patients With Rheumatoid Arthritis

**DOI:** 10.3389/fimmu.2021.744254

**Published:** 2021-10-15

**Authors:** Xin Wang, Hongxuan Fan, Yongle Wang, Xufang Yin, Guangying Liu, Chong Gao, Xiaofeng Li, Bin Liang

**Affiliations:** ^1^ Department of Rheumatology, The Second Hospital of Shanxi Medical University, Taiyuan, China; ^2^ Department of Cardiology, The Second Hospital of Shanxi Medical University, Taiyuan, China; ^3^ Department of Neurology, The First Hospital of Shanxi Medical University, Taiyuan, China; ^4^ Department of Pathology, Brigham and Women’s Hospital, Harvard Medical School, Boston, MA, United States

**Keywords:** T helper cell, atrial fibrillation, rheumatoid arthritis, Th1, Th17/Treg

## Abstract

Patients with rheumatoid arthritis (RA) have a significantly high risk of atrial fibrillation (AF). This study aimed to compare the absolute and relative changes in peripheral T cells in patients with RA who were also affected with and without AF. To help make an early diagnosis and prevent the initiation and progression of AF, the changes in the lymphocyte subsets were assessed in RA patients with and without AF. A propensity score matching (PSM) system (1:3) was used to perform a matched case-control study with 40 RA-AF cases and 120 RA controls. Changes in the erythrocyte sedimentation rate (ESR), C-reactive protein (CRP), anti-citrullinated peptide antibody (ACPA), and rheumatoid factor (RF) were examined. The percentage and absolute number of T, B, natural killer (NK), T helper (Th)1, Th2, Th17, and T-regulatory (Treg) cells in the peripheral blood of patients with and without RA-AF were determined using flow cytometry. Univariate and multivariate analyses were performed to determine the association between peripheral lymphocytes and RA-AF. Demographic data, ESR, CRP, ACPA, and the percentage, as well as the absolute value of B, NK, Th2, and Treg cells, showed no significant differences between the propensity score-matched groups of RA and RA-AF. Meanwhile, the absolute number and percentage of Th1 cells, the absolute number of Th17 cells, the ratio of Th1/Treg, Th17/Treg, and RF were significantly higher in patients with RA-AF than those in the control groups (P < 0.05). Univariate and multivariate logistic regression analyses also revealed that the percentage of Th1 cells, the absolute number of Th17 cells, and the ratio of Th1/Treg were associated with a significantly higher risk of AF. This PSM study demonstrated that the incidence of AF was higher in RA patients with Th cell immunological derangements.

## Introduction

Rheumatoid arthritis (RA), which affects 0.1%–2.0% of adults in industrialized countries, is an autoimmune, inflammatory, and multisystem disorder characterized by chronic synovitis, systemic inflammation, and autoantibodies, particularly including the rheumatoid factor (RF) and anti-citrullinated peptide antibody (ACPA) (1). Persistent or recurrent episodes of synovitis can lead to joint damage, decreased quality of life, and even disability. However, uncontrolled active RA not only causes joint damage but also can damage other tissues such as the brain, myocardium, and autonomic nerves. Cardiovascular disease (CVD) resulting from chronic inflammation plays a vital role in increasing the economic burden and mortality of patients with RA (2). Indeed, the prevalence of atrial fibrillation (AF) is significantly higher in patients with RA than in healthy individuals (3–5), suggesting that patients with RA-AF are characterized by destabilized diffuse myocardial electrophysiology induced by chronic inflammation (6).

In 2010, AF was the most common type of arrhythmia (estimated lifetime risk of 22%–26%), as it affected ~33.5 million individuals worldwide (7). More recently, a large national survey revealed that ~7.9 million people suffer from AF in China (8). AF is associated with substantial morbidity, reduced quality of life (QOL), and increased mortality due to the combination of altered hemodynamics, progressive atrial and ventricular mechanical dysfunction, heart failure, and thromboembolic complications; thus, AF is a major burden on the healthcare systems. However, the pathophysiology of AF is complex and not fully understood. Accumulating evidence indicates that its occurrence and progression are related to immune derangement. A meta-analysis has revealed a statistically significant increased risk of subsequent development of AF among patients with RA (9). Experimental evidence has demonstrated that RA increases AF susceptibility by inducing atrial remodeling (10). Although a relationship between RA and AF has been defined, early identification and anti-inflammatory therapy that can effectively prevent CVD have not been developed. This is mainly due to the long-term and insidious impact of chronic inflammation on the atrial substrate and the fact that AF has been largely ignored in patients with RA. Therefore, the clinical features of RA-AF need to be identified and validated to facilitate early diagnosis and implement protective actions to decelerate or alter the pathogenesis of AF in patients with RA. T cells are dominant contributors to the initiation and progression of RA, and their role in triggering and developing AF cannot be ignored (11, 12). Overall, immune derangement (especially T cells) in patients with RA results in AF.

Nevertheless, the absolute and relative changes in peripheral blood lymphocytes and T subsets in RA associated with AF (RA-AF) remain unclear. In the present study, we aimed to determine how the absolute numbers and ratios (%) of peripheral blood lymphocytes and T subsets differ between patients with RA and RA-AF and to establish a novel method for detecting early AF and improving the prognosis of these patients.

## Materials and Methods

### Patient Information

The research was completed, and the patients were retrospectively involved in the analysis, which was developed according to previous studies and physicians’ thinking in daily clinical practice. The study was approved by the Medical Ethics Committee of Shanxi Medical University, China [ID (2021): YX No. 035].

This retrospective case-control study used propensity score matching (PSM) to control for confounding factors. All data about patients were consecutively collected from the clinical database at the Department of Rheumatology, Second Hospital of Shanxi Medical University, between January 2016 and October 2020. Demographic, epidemiological, and medical information that met the RA classification standard of the American Society of Rheumatology (ACR) in 1987 was obtained from electronic medical and clinical laboratory records. The inclusion criteria comprised morning stiffness, arthritis in three or more joints, hand joints, symmetrical arthritis for at least 6 weeks (13), rheumatoid nodules, and changes in the serum rheumatoid factor and radiographical data. For classification purposes, patients who satisfied at least four of these seven criteria are considered to have RA. AF was defined according to the 2010 European Society of Cardiology guidelines (14) as surface ECG findings of absolutely irregular RR intervals and RR intervals that do not follow a repetitive profile, P-waves are not distinct on surface ECG, and usually variable atrial cycle lengths (interval between two atrial activations) <200 ms (>300 bpm). The exclusion criteria comprised valvular disease and/or mechanical heart valves; congenital heart disease; cardiomyopathy, malignant tumors; hyperthyroidism; active infection; kidney, liver, or other organ dysfunction; and long-term smoking and alcohol consumption. Screening according to the criteria selected, 747 patients with RA with information about age, sex, hypertension, coronary artery disease (CAD), stroke, erythrocyte sedimentation rate (ESR), rheumatoid factor (RF), ACPA, C-reactive protein (CRP), T-lymphocyte subsets, B-lymphocyte subsets, and natural killer (NK) cells were obtained from medical records. We finally included data from 40 and 120 patients who had RA with and without (control) AF determined by PSM (1:3).

### Detection of Peripheral Lymphocytes and CD4+ T-Cell Subsets by Flow Cytometry

The essential reagents were as follows: stimulin, ionomycin, Golgi blocker, fetal bovine serum, and RPMI 1640 medium (Sigma-Aldrich Corp., St. Louis, MO, USA). Absolute count microspheres-Trucount™ tubes, hemolysin, Multitest CD3-fluorescein isothiocyanate (FITC)/CD8-PE/CD45-PercP/CD4-APC kits, Multitest CD3-FITC/CD16+56-PE/CD45-PercP/CD19-APC kits, and monoclonal antibodies to CD4-FITC, IL-4-PE, IFN-c-APC, IL-17-PE, CD25-APC, and FOXP3-PE (Becton Dickinson and Co., Franklin Lakes, NJ, USA).

In brief, 20 μl of CD3FITC/CD8PE/CD45PercP/CD4APC antibody and 20 μl of CD3FITC/CD16+56-PE/CD45PercP/CD19APC antibody were vortex-mixed with 50 μl of fully anticoagulated blood in separate Trucount tubes, then placed at room temperature for 15 min. Thereafter, the contents of each tube were mixed and incubated with 450 μl of XFACS hemolysin at room temperature for 15 min for flow cytometry. We examined 15,000 cells obtained using the MultiSET™ software (Becton Dickinson and Co.).

### Culture and Detection of Th1/Th2/Th17 Cells

A mixture of phorbol myristate acetate (PMA; 10 μl, final concentration 30 ng/ml), 10 μl ionomycin (final concentration 750 ng/ml), 1 μl of BD GolgiStop™ (Becton Dickinson and Co.), and 80 μl of anticoagulated blood were incubated at 37°C for 5 h under a CO_2_ atmosphere. The cells were divided into tubes A and B and incubated with FITC-anti-human CD4 antibody for 30 min at room temperature in darkness. Fresh fixation/permeabilization solution was added and vortex-mixed, then the tubes were incubated at 4°C for 30 min in the dark.

Interleukin (IL)-4-PE and interferon (IFN)-c-APC were added to tube A, and anti-human IL-17-PE was added to tube B, then placed at room temperature for 30 min in the dark. The contents were washed with PBS, and cells were detected using the FACSCalibur flow cytometer (Becton Dickinson and Co.).

### Culture and Detection of Treg Cells

Anticoagulated blood (80 μl) was incubated with CD4-FITC and CD25-APC at room temperature for 30 min in darkness, followed by 1 ml of freshly prepared fixation/permeabilization solution at 4°C for 30 min in darkness. Cells were stained with anti-human forkhead box P3 (FOXP3) antibody at room temperature for 30 min in the dark, washed with PBS, and detected using flow cytometry.

### Flow Cytometry Assays

Cells were detected by flow cytometry within 24 h. Th (CD4+) cells were distinguished according to the forward and side scatter values. Relative ratios were analyzed using the CellQuest™ software (Becton Dickinson and Co.). The absolute numbers of cells in the subgroups were calculated as the absolute cell counts of the ratio (%) between the positive cells in each subgroup multiplied by the absolute number of CD4+ T cells/μl.

### Statistical Analysis

The normality of continuous variable distribution was evaluated using Shapiro–Wilk test. Normally distributed data were expressed as means with standard deviation (SD). Non-normally distributed data were expressed as medians with interquartile ranges (IQRs). Categorical variables were described as frequencies and ratios (%). The normally distributed values were compared using unpaired Student’s t-tests between RA groups with and without AF. The non-normally distributed values were compared using Wilcoxon rank-sum or Mann–Whitney tests between RA groups with and without AF. Potential predictors of AF were initially investigated by univariate logistic regression proportional hazards regression analysis, followed by multivariate analysis to identify independent predictors and their power. All values with P < 0.05 were regarded as statistically significant.

### Propensity Score-Matched Analysis

We applied PSM to minimize bias in the selection of patients who had RA with and without AF. Propensity scores were constructed for each participant using the confounding categorical variables that influenced the incidence of AF: age, sex, CAD, hypertension, and stroke. We applied optimal PSM to generate a cohort of 40 matched pairs. Rubin recommends a B value (standardized difference in mean propensity scores) of <25 for samples to be considered sufficiently balanced and the variance ratio in the propensity score between the treated and comparison groups.

## Results

### General Features of Patients With Rheumatoid Arthritis and Atrial Fibrillation

**Figure 1:**
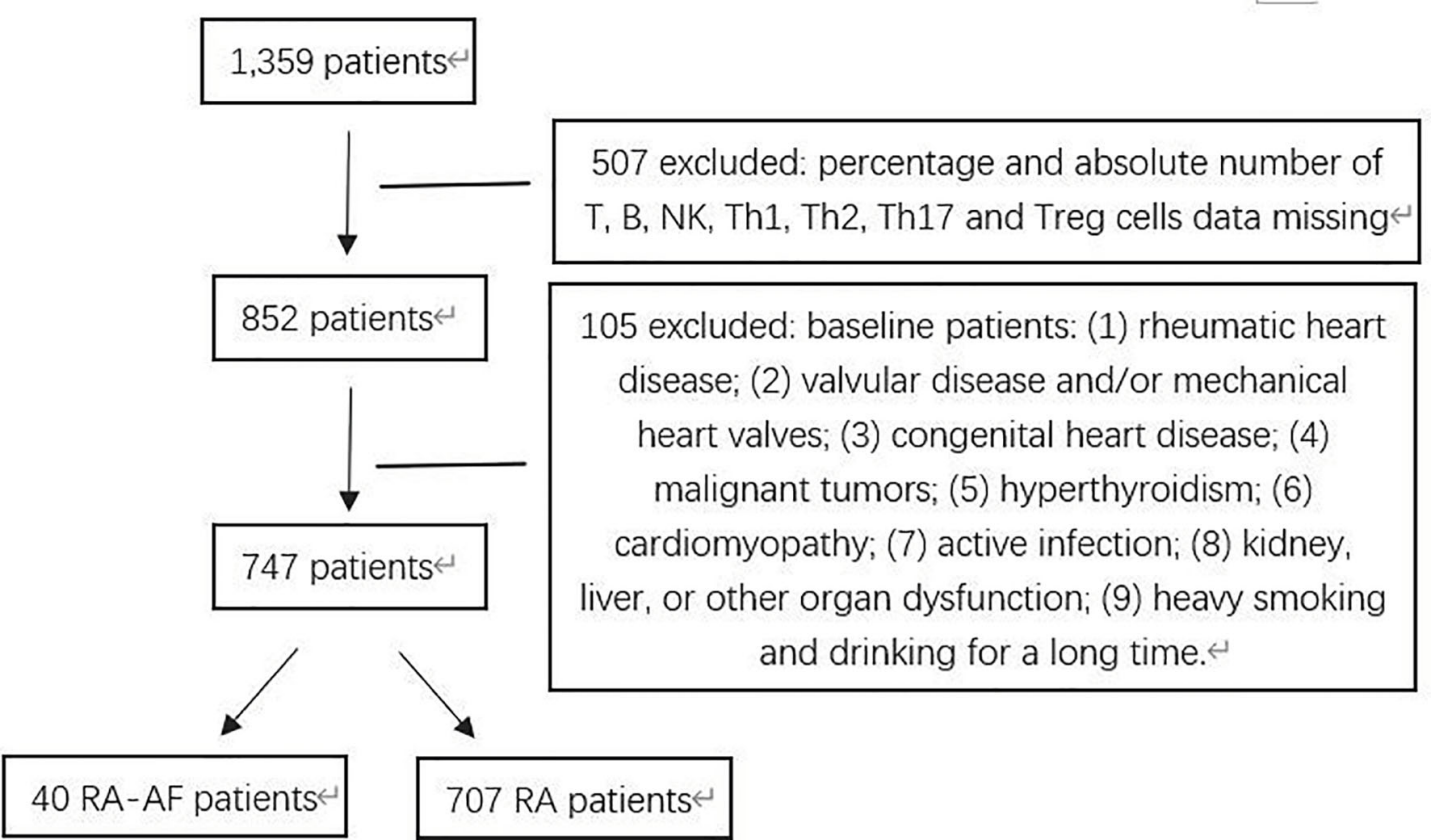
Flowchart of patients with rheumatoid arthritis (RA) and RA presenting atrial fibrillation (AF) enrolled in this study.

**Figure 1** shows a flowchart of patient enrollment, and **Table 1** shows the characteristics of the patients with RA and controls before and after PSM. Age, sex, CAD, hypertension, and stroke did not significantly differ between the groups (P > 0.05) after PSM (**Table 1**). The inflammatory markers, ESR, CRP, and ACPA, did not significantly differ between the two groups (P > 0.05), but levels of RF were significantly higher in patients with RA-AF (**Table 3**).

**Table 1:** Baseline characteristics of patients with RA (control) and with RA and AF.

Characteristics	Before PSM			After PSM (1:3)		
	RA-AF (n = 40)	Control (n = 707)	P	RA-AF (n = 40)	Control (n = 120)	P
Age (years)	65.67 ± 9.61	59.56 ± 9.54	0.000088	65.67 ± 9.61	67.38 ± 9.41	0.324
Male sex	18 (45.00%)	215 (30.41%)	0.052658	18 (45.00%)	41 (34.17%)	0.219
Hypertension	17 (42.50%)	200 (28.29%)	0.054096	17 (42.50%)	33 (27.50%)	0.076
CAD	14 (35.00%)	33 (4.67%)	<0.001	14 (35.00%)	32 (26.67%)	0.313
Stroke	10 (25.00%)	32 (4.53%)	<0.001	10 (25.00%)	20 (16.67%)	0.242

Data are shown as means ± SD or medians with interquartile ranges (IQRs). AF, atrial fibrillation; CAD, coronary artery disease, PSM, propensity score matching; RA, rheumatoid arthritis.

### Comparison of Peripheral Lymphocyte Subsets Between RA-AF and RA Groups

**Figure 2:**
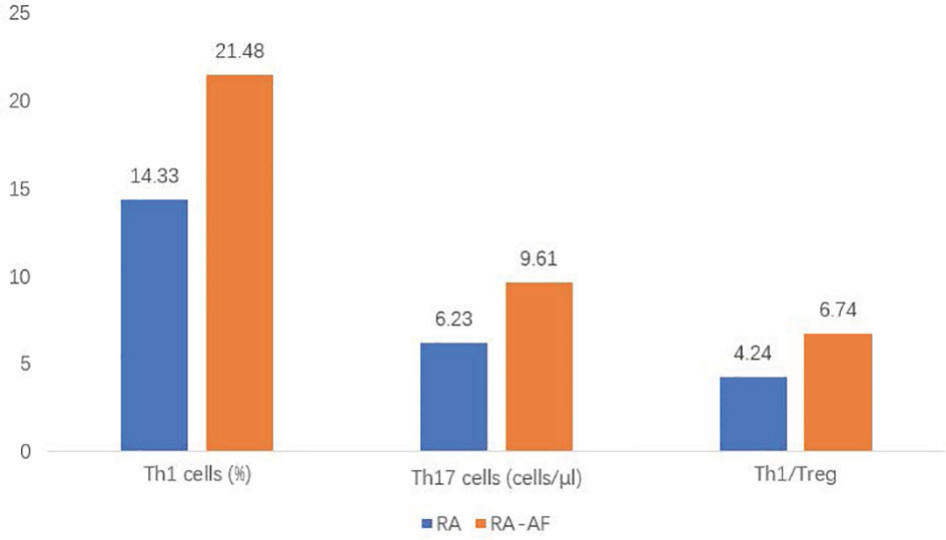
Comparison of proportions of T helper (Th)1, Th17, and Th1/T-regulatory (Treg) cells between rheumatoid arthritis (RA) and RA-atrial fibrillation (AF) groups.

For the peripheral lymphocyte subsets, the ratios (%) of T-helper (Th)1 cells, ratios of Th1/Th17, and Th17/Treg cells, as well as the absolute numbers of Th1 and Th17 cells were higher in the RA-AF than those in the RA group (**Figure 2**). However, levels of B, CD8+ T, and NK cells did not significantly differ between the two groups (**Tables 2**, **3**).

**Table 2:** Absolute numbers and ratios (%) of peripheral lymphocyte subsets in patients with RA-AF and RA.

	RA-AF (n = 40)	RA (n = 120)	P
T cells (%)	68.58 ± 10.28	70.26 ± 9.67	0.352
B cells (%)	12.33 ± 6.25	10.54 ± 6.01	0.108
CD4+ T cells (%)	39.01 ± 10.49	41.75 ± 8.50	0.099
CD8+ T cells (%)	26.31 ± 10.07	25.47 ± 8.97	0.619
CD3-CD56+ NK cells (%)	16.98 ± 10.22	16.94 ± 9.13	0.984
T+B+ NK (%)	97.84 ± 1.00	97.73 ± 1.34	0.636
Th1 cells (%)	21.48 ± 14.30	14.33 ± 9.82	0.007
Th2 cells (%)	1.25 ± 0.57	1.52 ± 1.75	0.483
Th17 cells (%)	1.26 ± 0.75	3.28 ± 8.77	0.284
Treg cells (%)	4.31 ± 1.99	5.13 ± 4.18	0.234
T cells/μl	1,332.16 ± 668.06	1,220.31 ± 508.06	0.269
B cells/μl	240.58 ± 208.71	184.55 ± 127.80	0.045
CD4+ T cells/μl	778.36 ± 478.48	724.11 ± 312.20	0.958
CD8+ T cells/μl	530.50 ± 351.81	473.72 ± 277.56	0.298
CD3-CD56+ NK cells/μl	329.29 ± 242.60	289.26 ± 185.54	0.277
T+B+ NK cells/μl	1,976.28 ± 1062.04	1,736.13 ± 663.51	0.243
Th1 cells/μl	156.13 ± 129.23	101.33 ± 95.39	0.006
Th2 cells/μl	9.51 ± 5.59	7.53 ± 6.02	0.068
Th17 cells/μl	9.61 ± 7.34	6.23 ± 5.04	0.012
Treg cells/μl	32.29 ± 22.03	27.00 ± 18.08	0.132

Data are shown as means ± SD. Normal values were compared using paired and unpaired Student’s t-tests, and non-normally distributed values were compared using Wilcoxon rank-sum or Mann–Whitney tests. AF, atrial fibrillation; NK, natural killer; RA, rheumatoid arthritis; Th, T helper; Treg, T regulatory.

**Table 3:** Ratios of peripheral lymphocyte subsets and inflammatory biomarkers in patients with RA-AF and RA.

	RA-AF (n = 40)	RA (n = 120)	P
Th/Ts	1.76 ± 1.05	1.93 ± 1.44	0.499
Th1/Th2	20.55 ± 17.00	23.50 ± 71.93	0.798
Th17/Treg	0.35 ± 0.28	0.27 ± 0.19	0.211
Th1/Treg	6.74 ± 7.45	4.24 ± 3.97	0.084
Th2/Treg	0.35 ± 0.23	0.30 ± 0.20	0.221
B cell/Treg	10.04 ± 9.20	11.14 ± 17.12	0.699
NK cell/Treg	14.08 ± 16.11	21.49 ± 46.23	0.323
ESR	54.32 ± 40.63	55.76 ± 36.27	0.732
CRP	35.32 ± 48.60	32.58 ± 34.84	0.958
ACPA	536.48 ± 556.97	563.88 ± 612.50	0.604
RF	375.62 ± 425.70	269.70 ± 470.77	0.005

ACPA, anti-citrullinated peptide antibody; AF, atrial fibrillation; CRP, C-reactive protein; ESR, erythrocyte sedimentation rate; NK, natural killer; RA, rheumatoid arthritis; RF, rheumatoid factor; Th, T helper; Treg, T regulatory.

### Univariate and Multivariate Analyses of Factors Associated With Rheumatoid Arthritis-Atrial Fibrillation

The results of the univariate logistic analysis showed that the ratios of Th1 cells, Th1:Th17, and Th17:Treg cells, as well as the absolute numbers of Th1 and Th17 cells were significantly associated with RA-AF. We applied multivariate logistic analysis to exclude the effects of age, sex, CAD, hypertension, and stroke. The results showed that the ratio of Th1 cells [odds ratio (OR), 1.05; 95% confidence interval (CI), 1.02–1.08, P = 0.0029], absolute number of Th17 cells (OR, 1.11; 95% CI, 1.03–1.19, P = 0.0044), and the ratio of Th1/Treg (OR, 1.08; 95% CI, 1.00–1.16, P = 0.0414) were associated with RA-AF (**Table 4**).

**Table 4:** Univariate and multivariate analyses of factors associated with RA-AF.

	Non-adjusted	Model I	Model II
	OR (95% CI) P	OR (95% CI) P	OR (95% CI) P
T cells/μl	1.00 (1.00, 1.00) 0.2687	1.00 (1.00, 1.00) 0.2831	1.00 (1.00, 1.00) 0.2206
T cells (%)	0.98 (0.95, 1.02) 0.3514	0.98 (0.94, 1.01) 0.2302	0.97 (0.94, 1.01) 0.1502
Th1 cells/μl	1.00 (1.00, 1.01) 0.0094	1.00 (1.00, 1.01) 0.0090	1.00 (1.00, 1.01) 0.0160
Th1 cells (%)	1.05 (1.02, 1.08) 0.0012	1.05 (1.02, 1.09) 0.0012	1.05 (1.02, 1.08) 0.0027
Th2 cells/μl	1.05 (1.00, 1.12) 0.0728	1.06 (1.00, 1.13) 0.0462	1.06 (1.00, 1.13) 0.0664
Th2 cells (%)	0.86 (0.61, 1.19) 0.3594	0.87 (0.62, 1.23) 0.4366	0.86 (0.59, 1.26) 0.4323
Th17 cells/μl	1.10 (1.03, 1.17) 0.0039	1.11 (1.04, 1.19) 0.0030	1.11 (1.03, 1.18) 0.0046
Th17 cells (%)	0.92 (0.81, 1.05) 0.2406	0.93 (0.81, 1.06) 0.2508	0.93 (0.81, 1.06) 0.2866
Treg cells/μl	1.01 (1.00, 1.03) 0.1388	1.01 (1.00, 1.03) 0.1181	1.01 (1.00, 1.03) 0.1252
Treg cells/μl	0.92 (0.80, 1.06) 0.2459	0.93 (0.81, 1.06) 0.2758	0.93 (0.81, 1.08) 0.3447
Th1/Th2	1.00 (0.99, 1.01) 0.8000	1.00 (0.99, 1.01) 0.8005	1.00 (0.99, 1.01) 0.8480
Th17/Treg	4.84 (1.02, 23.05) 0.0478	5.31 (1.10, 25.66) 0.0379	4.29 (0.85, 21.75) 0.0785
Th1/Treg	1.09 (1.02, 1.16) 0.0159	1.09 (1.01, 1.17) 0.0194	1.08 (1.00, 1.16) 0.0374
Th2/Treg	2.69 (0.54, 13.33) 0.2258	2.63 (0.53, 13.19) 0.2389	2.21 (0.41, 11.77) 0.3534

Data are shown as means (SD) or ratios. None, non-adjusted model. Model I adjusted for sex and age; Model II adjusted for sex, age, coronary artery disease, hypertension, and stroke. AF, atrial fibrillation; OR, odds ratio; RA, rheumatoid arthritis; Th, T helper; Treg, T regulatory.

## Discussion

**Figure 3:**
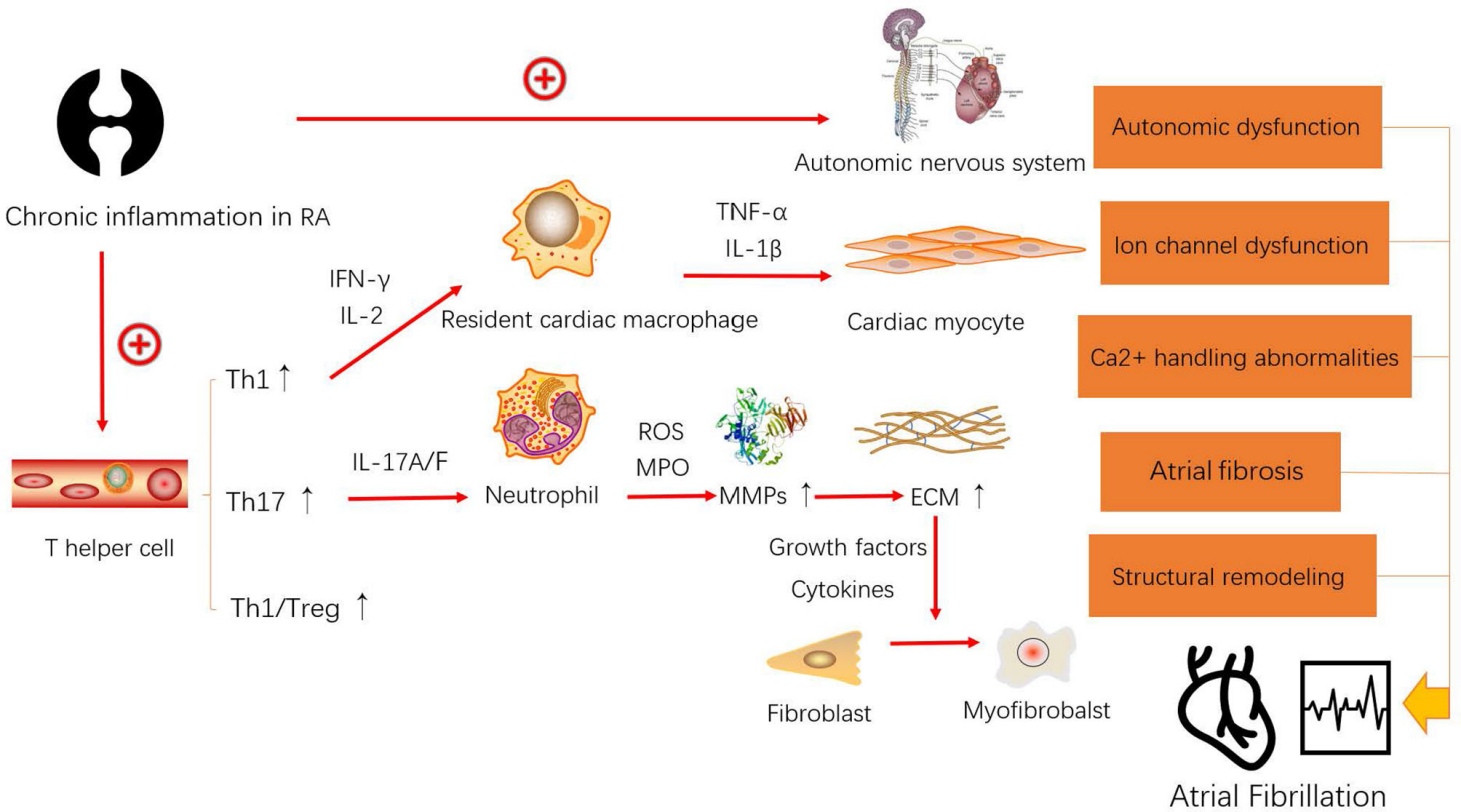
Model of the possible mechanism leading to the occurrence and development of atrial fibrillation (AF) caused by rheumatoid arthritis (RA).

RA is well known to increase the risk of cardiovascular events. Autopsy findings have revealed that many patients with RA had also been diagnosed with cardiac amyloidosis (15). Comprehensive cardiac magnetic resonance imaging has uncovered myocardial abnormalities in patients with RA who were asymptomatic for cardiac disease (16). Although the relationship between RA and cardiovascular events (17), such as atherosclerosis (18) and sudden cardiac death (19), has been investigated in detail, the role of RA in AF remains unclear. RA is not limited to the local synovial joints but can affect organs and vessels *via* peripheral blood containing CD4+ T cells, which activate numerous inflammatory cells that spread chronic inflammation throughout the body. Naive CD4+ T cells differentiate into various Th cell subsets and play various roles in autoimmune diseases. The immune response mechanism is unclear and complex in RA-AF. There is accumulating evidence that suggests that the immune response and AF are closely related (12, 20). Whether the immune response is a cause or an effect of RA-AF remains inconclusive. As a functional unit of the immune system, immune cells, especially CD4+ T cells, might play vital roles in the immunological pathogenesis of RA-AF (**Figure 3**).

Classically, Th1 cells that produce IFN-γ and IL-2 might play a substantial role in the synovial fluid and peripheral blood during the development of RA. The peripheral blood of patients with RA contains citrulline-specific T cells, of which ~40% of the samples are positive for the Th1 marker chemokine receptor 3 (CXCR3) (21), and plasma concentrations of IL-2 and IFN-γ, which are also biomarkers of Th1 cells, are significantly elevated in patients with AF (13, 22, 23). Among them, IL-2 activates T lymphocytes and stimulates the synthesis of TNF-α and IFN-γ, both of which exert biological effects that might promote atrial remodeling through macrophages *via* stimulating cytokine secretion. Thus, the major functions of Th1 cells in AF are to maximize the efficacy of macrophages and promote the inflammatory process.

Th17 cells are considered a contributing factor in the development of AF. IL-17A produced mainly by Th17 cells is a key mediator of the activation, recruitment, and migration of neutrophils. Subsequently, the pathogenic role of Th17 cells that produce IL-17A has intrigued rheumatologists. The proportion of IL-17-positive CD4+ T cells (Th17 cells) is higher in peripheral blood mononuclear cells from patients with RA compared with healthy controls, and their proportion correlates with systemic disease activity at both the onset and during the progression of RA (24, 25). Th17 cells might be involved in the pathogenesis of AF, as elevated plasma levels of Th17-associated cytokines were independently associated with the increased risk of AF, further suggesting that Th17 cells are involved in the pathogenesis of AF (26). Interleukin-17A contributes to the development of AF by promoting inflammation and cardiac fibrosis (27) and affecting protein kinase C (PKC)-β and Erk 1/2 phosphorylation, as well as nuclear factor (NF)-κB activation in fibroblasts from model mice (28). Elevated IL-17A levels are related to AF pathology that is mediated by neutrophils (29).

The core treatment of RA can complete the conversion of Th1/Th2 to Th17/Treg cells (30). However, others have also focused on Th1/Treg cells (31). Nowadays, many studies have revealed that Th1/IL-12 and Treg/IL-10 have a very close activation relationship (32, 33). IL-12 produced by cells of the innate immune system and by B cells plays a vital role in regulating naive T cells to differentiate into Th1 cells (33). Furthermore, the increase of IL-12 is highly relevant with AF by causing cardiac fibrosis (34). Meanwhile, IL-10 produced by Treg cells is a potent anti-inflammatory cytokine (33). A recent study has suggested that IL-10(-592A/C) polymorphism may have a prominent association with postoperative AF (35). Therefore, we reasonably speculate that the increase of Th1/Treg may associate with the development of RA-AF. **Figure 3** shows the mechanism through which RA influences the occurrence and development of AF. Chronic inflammation in RA causes autonomic nervous system (ANS) dysfunction. Approximately 60%–80% of the patients with RA present ANS dysfunction as a sign of cardiovascular reflex impairment and altered heart rate variability, which indicates reduced and elevated cardiac parasympathetic and sympathetic activity, respectively (36). A disabled ANS is a considerable reason for triggering ectopic pacemakers in AF. Furthermore, elevated Th1 cells produce more IFN-γ to activate resident cardiac macrophages (22), which in turn produce tumor necrosis factor (TNF)-α and IL-1β, both of which can damage cardiac myocytes (37). There is considerable evidence supporting that macrophages play a core role in promoting electrical and structural remodeling by altering Ca^2+^ handling, shortening the action potential duration, reducing Cx40 and Cx43, causing fibrosis, and generating cardiac myocyte apoptosis and myolysis during the progress of AF (38). Th17 cells exert IF-17A/F to activate neutrophils. A theory has been introduced to explain the association between neutrophils and atrial fibrosis in AF (39). Polymorphonuclear neutrophils (PMNs) can infiltrate the myocardial interstitium and produce myeloperoxidase (MPO) and reactive oxygen species (ROS) to help convert pro-matrix metalloproteins (MMPs) to MMPs (40). Increased levels of MMPs result in the degradation of the extracellular matrix (ECM), which results in the release of growth factors [such as transforming growth factor (TGF)-β] and cytokines (such as IL-6), to assist the conversion of fibroblasts into myofibroblasts (41). Therefore, PMNs cause atrial fibrosis and progress to structural remodeling. In conclusion, elevated Th1 and Th17 levels in the peripheral blood of patients with RA putatively cause AF through overactivated resident cardiac macrophages and PMNs.

The effects of Th1 and Th17 and the anti-inflammatory effects of Tregs in RA were confirmed. We found an imbalance in CD4+ T cells in the peripheral blood of patients with RA with and without AF. This imbalance of CD4+ T cells leaning toward an unfavorable outcome was more obvious in patients with RA-AF. Thus, we concluded that a higher proportion of Th1 cells, the absolute number of Th17 cells, and the Th1/Treg ratios in the peripheral blood of patients with RA can indicate a tendency to develop AF. This suggests that RA is an important player in the development of AF, which might be associated with disordered T cells and harmful cytokines. Cardiovascular mortality is significantly associated with CD4+ CD28null cells in patients with AF [adjusted hazard ratio (HR), 1.59; 95% CI, 1.13–2.24, P = 0.008] (42). These cells can predict the development of postoperative AF after cardiac surgery (43). A risk management system should be developed to prevent RA-AF. For patients with RA at a high risk of developing AF, timely anti-inflammatory treatment should be administered to maintain a steady state of Th1, Th17, and Th1/Treg cells.

We revealed that increased frequencies of Th1, Th17, and Th1/Treg cells in the peripheral blood of patients with RA were associated with the development of AF. Our findings suggested that the immune response is involved in the pathogenesis of RA-AF, and that downregulated Th1, Th17, and Th1/Treg cells could be potential therapeutic targets for developing effective treatments against RA-AF. However, the mechanisms through which elevated Th1, Th17, and Th1/Treg cells in the peripheral blood remodel the cardiac tissues and cause AF await further investigation.

## Data Availability Statement

The original contributions presented in the study are included in the article/supplementary material. Further inquiries can be directed to the corresponding authors.

## Ethics Statement

The study was approved by the Medical Ethics Committee of Shanxi Medical University ID: (2021) YX No. 035. Written informed consent for participation was not required for this study in accordance with the national legislation and the institutional requirements.

## Author Contributions

BL and XW, study design. YW and HF, data analysis. HF, article drafting. XY, GL, and CG, data collection. XL, article revision. BL, review and final approval. All authors contributed to the article and approved the submitted version.

## Funding

This work was supported by the National Natural Science Foundation of China (grant number 81970391) and the Excellent Youth Foundation of Shanxi Province (grant number 201901D211504).

## Conflict of Interest

The authors declare that the research was conducted in the absence of any commercial or financial relationships that could be construed as a potential conflict of interest.

## Publisher’s Note

All claims expressed in this article are solely those of the authors and do not necessarily represent those of their affiliated organizations, or those of the publisher, the editors and the reviewers. Any product that may be evaluated in this article, or claim that may be made by its manufacturer, is not guaranteed or endorsed by the publisher.
